# Assessing Liver Fibrosis in Chronic Hepatitis B: Liver Biopsy or Non-Invasive Fibrosis Markers?

**DOI:** 10.3390/jcm14228164

**Published:** 2025-11-18

**Authors:** Deniz Borcak, Zuhal Yesilbag, Yusuf Emre Ozdemir, Adile Sevde Demir, Esra Salim Dogdas, Aysegul Inci Sezen, Esra Canbolat Unlu, Sevtap Senoglu, Hayat Kumbasar Karaosmanoglu, Kadriye Kart Yasar

**Affiliations:** 1 Department of Infectious Diseases and Clinical Microbiology, Bakirkoy Dr. Sadi Konuk Training and Research Hospital, 34147 Istanbul, Turkey; 2 Department of Infectious Diseases and Clinical Microbiology, Taksim Training and Research Hospital, 34433 Istanbul, Turkey

**Keywords:** APRI, non-invasive markers, FIB-4, GPR, hepatitis B, liver fibrosis, s-index

## Abstract

**Background/Objectives**: The accurate evaluation of the fibrosis stage is critical for improving chronic hepatitis B (CHB) management and patient outcomes. This study aimed to compare the diagnostic accuracy of non-invasive fibrosis markers with liver biopsy for detecting significant, advanced fibrosis and cirrhosis. We further investigated the diagnostic performance of non-invasive markers according to HBeAg status to provide further insight into their clinical utility across patient subgroups. **Methods**: This single-center retrospective study included 536 treatment-naive patients with CHB who underwent liver biopsy. Patients were categorized into four groups according to the fibrosis stage: “no significant fibrosis” (F0–F2), “significant fibrosis” (F3–F6), “advanced fibrosis” (F4–F6), and “cirrhosis” (F5–F6). AAR, AAPRI, APRI, API, FIB-4, GPR, and S–index were compared among these groups. **Results**: In total, 536 treatment-naïve patients were analyzed (63.2% male; mean age 44.8 ± 12.9 years), of whom 25.4% were HBeAg-positive. API, FIB 4, GPR, and S-Index showed good performance (area under the curve [AUC] ≥ 0.8–0.9) in defining advanced fibrosis (≥F4), AAPRI, AAR, API, and APRI showed good performance ([AUC] < 0.700) in defining cirrhosis. The analysis showed that GPR had the highest AUC for ≥F3 (0.719) and ≥F4 (0.838), while FIB 4 had the highest AUC for cirrhosis (0.865). **Conclusions**: These findings highlight the value of non-invasive markers as inexpensive and easily applicable methods for clinicians in assessing the stage of liver fibrosis. The integration of these scores into the routine monitoring of chronic hepatitis B patients is expected to expand, enhancing clinical decision-making and reducing the necessity for liver biopsies.

## 1. Introduction

Liver fibrosis is a major risk factor for the progression of chronic hepatitis B infection (CHB) to liver cirrhosis and hepatocellular carcinoma [1]. Accurate evaluation of fibrosis stage and timely initiation of antiviral treatment are critical for improving disease management and patient outcomes [2]. Liver biopsy remains the gold standard method for staging liver fibrosis; however, its clinical use is limited by several important drawbacks. It is an invasive and costly intervention that carries the risks of severe complications (0.53–1.1%) and mortality (0.01–0.11%) [3,4,5]. In addition, sampling errors, contraindications in some cases, and intra- and inter-observer variability may reduce its reliability and clinical applicability. The procedure lacks reproducibility and requires professional histological examination. Moreover, many patients are reluctant to undergo liver biopsy frequently due to psychological reasons, such as anxiety. While conventional blood tests, including serum alanine aminotransferase (ALT), are useful for assessing disease activity, they are poor predictors of liver fibrosis when used alone [6,7]. These limitations have encouraged the search for reliable non-invasive techniques that may serve as safe and practical alternatives in clinical practice.

Non-invasive markers enable clinicians to distinguish patients with mild fibrosis from those with more advanced stages, such as significant fibrosis or cirrhosis, without the risks associated with liver biopsy [8]. Although these markers are often used in the diagnosis of cirrhosis, evidence regarding their use and accuracy in identifying significant and advanced fibrosis has not yet been well established. The Aspartate Aminotransferase-to-Platelet Ratio Index (APRI) and Fibrosis-4 (FIB-4) have been included in worldwide guidelines for HBV management and are increasingly being utilized in clinical decision-making [9,10]. Moreover, among non-invasive markers, APRI and FIB-4 have been the most extensively studied and validated in clinical research for predicting advanced fibrosis and cirrhosis [11,12]. The data regarding the other non-invasive markers are relatively scarce. Therefore, this study aimed to contribute to the existing literature by evaluating and comparing the diagnostic performance of seven non-invasive fibrosis markers against liver biopsy findings in patients with CHB. Our study provides a comprehensive analysis of the different stages of liver disease, including significant fibrosis, advanced fibrosis, and cirrhosis. We also investigated the diagnostic performance of non-invasive markers according to HBeAg status, offering new insights into their clinical utility in different patient subgroups.

## 2. Materials and Methods

### 2.1. Study Design

This retrospective single-center study was conducted between January 2015 and January 2023 at a 507-bed tertiary hospital in Istanbul, Turkey, and included treatment-naive patients with CHB who underwent liver biopsy. Demographic, laboratory, radiological, and histopathological data were retrieved from electronic records.

### 2.2. Exclusion Criteria

Patients with a history of significant alcohol use (≥30 g/day for men and ≥20 g/day for women) were excluded, as well as those using drugs known to cause fatty liver disease. Other exclusion criteria included the presence of chronic liver diseases such as autoimmune hepatitis, primary biliary cholangitis, drug-induced liver injury, hemochromatosis, Wilson’s disease, or coinfection with other viral hepatitis agents (hepatitis C or D). Body mass index (BMI) was calculated as weight (kg) divided by height squared (m^2^), and patients with a BMI ≥ 30 kg/m^2^ were also excluded from the study.

### 2.3. Definitions

Definitions of HBeAg-negative and HBeAg-positive chronic hepatitis B (CHB) and HBV infection were based on the 2025 guidelines from the European Association for the Study of the Liver [13]. Histopathological evaluation of liver biopsies was assessed using the Ishak-modified Histology Activity Index (HAI) scoring system before the initiation of antiviral treatment. The ISHAK score assesses the degree of fibrosis on a scale from F0 to F6 [14]. Patients were classified into four groups based on the fibrosis stage: “no significant fibrosis” (F0–F2), “significant fibrosis” (F3–F6), “advanced fibrosis” (F4–F6), and “cirrhosis” (F5–F6). The non-invasive markers used to assess hepatic fibrosis were calculated using the following formulas: AAPRI = AAR/PLT (10^9^/L); AAR = AST/ALT; API = sum of the points from the age and PLT group [15]; APRI = (AST/upper limit of normal for AST)/PLT (10^9^/L) × 100; FIB-4 = (Age [years] × AST)/(PLT [10^9^/L] × √ALT); GPR = (GGT/upper limit of normal for GGT)/PLT (10^9^/L) × 100; S–index = (1000 × GGT)/(PLT [10^9^/L] × Albumin^2^) [16]. The area under the receiver operating characteristic curve (AUC) was used to evaluate the diagnostic performance of non-invasive markers and classified as poor (<0.7), moderate (≥0.7–0.8), good (≥0.8–0.9), and excellent (≥0.9) [17].

### 2.4. Statistical Analysis

Statistical analyses were performed using SPSS v25.0 for Windows (SPSS Inc., Chicago, IL, USA). Descriptives were presented as mean ± SD, frequency, median (IQR), and percentage values. The normality of continuous variables was tested with the Kolmogorov–Smirnov test. Categorical variables were compared using the Chi-Square and Fisher’s Exact test. Student’s *t*-test was used for normally distributed continuous variables, and the Mann–Whitney U test was used for non-normally distributed continuous variables. The Youden index method was applied to calculate the new cut-off values for the non-invasive fibrosis markers. Results with *p* < 0.05 were considered statistically significant.

## 3. Results

### 3.1. Patient Baseline Characteristics

This study included 536 treatment-naive patients, of whom 339 (63.2%) were male, with a mean age of 44.8 ± 12.91 years (range 19–84). Among all patients, 136 (25.4%) were HBeAg-positive. The distribution of fibrosis stages was as follows: F0 (*n* = 14, 2.6%), F1 (*n* = 67, 12.5%), F2 (*n* = 364, 67.9%), F3 (*n* = 64, 11.9%), F4 (*n* = 13, 2.4%), F5 (*n* = 3, 0.6%), and F6 (*n* = 11, 2.1%). Overall, non-significant fibrosis was identified in 445 patients (83%), significant fibrosis in 91 (17%), advanced fibrosis in 27 (5%), and cirrhosis in 14 (2.6%). There was a statistically significant difference between the fibrosis-degree groups in terms of age, AST, albumin, platelet count, and gender (*p* < 0.001). Demographic characteristics and laboratory findings of the patients according to fibrosis stages are presented in Table 1.

The mean values for AAPRI, AAR, API, APRI, FIB-4, GPR, and S-index were 0.004 ± 0.002, 0.822 ± 0.343, 1.880 ± 1.781, 0.786 ± 0.888, 1.338 ± 1.426, 0.482 ± 0.617, and 0.134 ± 0.322, respectively. The median values of AAPRI, API, APRI, FIB-4, GPR, and S-Index differed significantly between the non-significant fibrosis and significant fibrosis groups (*p* < 0.001) and between the non-cirrhotic and cirrhotic groups (*p* < 0.001). The comparison of the non-invasive markers between fibrosis stages and cirrhosis status is presented in Table 2.

### 3.2. Prediction of Significant Fibrosis (≥F3)

**Figure 1:**
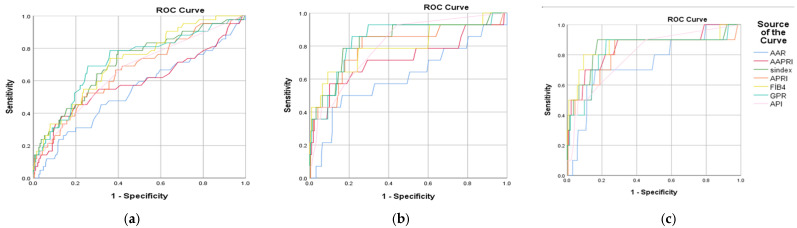
Receiver operating characteristic curves of non-invasive fibrosis markers (AAR, AAPRI, APRI, API, FIB-4, GPR, and S–index) for the identification of (**a**) significant fibrosis, (**b**) advanced fibrosis, and (**c**) cirrhosis.

The ROC curve analysis for predicting ≥F3 showed AUC values for AAPRI, AAR, API, APRI, FIB-4, GPR, and S-Index were 0.590 (95% CI: 0.48–0.69; *p* < 0.001), 0.540 (95% CI: 0.43–0.64; *p* = 0.421), 0.661 (95% CI: 0.56–0.75; *p* < 0.001), 0.664 (95% CI: 0.57–0.75; *p* < 0.001), 0.717 (95% CI: 0.63–0.80; *p* < 0.001), 0.719 (95% CI: 0.63–0.80; *p* < 0.001), and 0.712 (95% CI: 0.62–0.80; *p* < 0.001), respectively (Figure 1).

Among these, AAPRI, AAR, API, and APRI showed poor accuracy, whereas FIB-4, GPR, and S-Index demonstrated moderate accuracy. Notably, in HBeAg-positive patients, FIB-4 0.817 (95% CI: 0.68–0.94; *p* = 0.004) and S-Index 0.803 (95% CI: 0.68–0.9; *p* = 0.004) showed good performance, whereas GPR 0.713 (95% CI: 0.60–0.81; *p* < 0.001) and S-Index 0.702 (95% CI: 0.59–0.80; *p* < 0.001) showed moderate performance in HBeAg-negative patients. The cut-off values calculated for AAPRI, AAR, API, APRI, FIB-4, GPR, and S-Index were 0.004, 0.805, 1.5, 0.555, 1.17, 0.445, and 0.075, respectively. The diagnostic performance of non-invasive fibrosis markers and the cut-off values with sensitivities and specificities for significant fibrosis are presented in Table 3.

### 3.3. Prediction of Advanced Fibrosis (≥F4)

The ROC curve analysis for advanced fibrosis (≥F4) demonstrated that the area under the curve (AUC) values for AAPRI, AAR, API, APRI, FIB-4, GPR, and S-Index were 0.723 (95% CI: 0.54–0.89; *p* = 0.005), 0.540 (95% CI: 0.43–0.64; *p* = 0.421), 0.805 (95% CI: 0.69–0.91; *p* < 0.001), 0.797 (95% CI: 0.65–0.94; *p* < 0.001), 0.800 (95% CI: 0.65–0.94; *p* < 0.001), 0.838 (95% CI: 0.71–0.96; *p* < 0.001), and 0.836 (95% CI: 0.71–0.96; *p* < 0.001), respectively (Figure 1b). The API, FIB-4, GPR, and S-Index showed good performance; APRI and AAPRI demonstrated moderate performance, whereas AAR showed a poor performance. Among HBeAg-positive patients, GPR 0.873 (95% CI: 0.77–0.97; *p* = 0.006) and S-Index 0.850 (95% CI: 0.73–0.96; *p* = 0.011) showed good performance, and FIB-4 0.754 (95% CI: 0.54–0.96; *p* = 0.063) showed moderate performance. In HBeAg-negative patients, FIB-4 0.848 (95% CI: 0.66–1.00; *p* < 0.001), S-Index 0.826 (95% CI: 0.63–1.00; *p* = 0.001), and APRI 0.822 (95% CI: 0.62–1.00; *p* = 0.001) showed good performance. The cut-off values calculated for AAPRI, AAR, API, APRI, FIB-4, GPR, and S-Index were 0.004, 0.805, 2.50, 0.835, 1.445, 0.525, and 0.125, respectively. The diagnostic performance of non-invasive fibrosis markers and the cut-off values with sensitivities and specificities for advanced fibrosis are presented in Table 4.

### 3.4. Prediction of Cirrhosis

The ROC curve analysis for predicting cirrhosis indicated that the area under the curve (AUC) values for AAPRI, AAR, API, APRI, FIB-4, GPR, and S-Index were 0.850 (95% CI: 0.70–0.99; *p* < 0.001), 0.748 (95% CI: 0.58–0.91; *p* = 0.008), 0.805 (95% CI: 0.69–0.91; *p* < 0.001), 0.811 (95% CI: 0.63–0.98; *p* < 0.001), 0.865 (95% CI: 0.70–1.00; *p* < 0.001), 0.826 (95% CI: 0.66–0.99; *p* < 0.001), and 0.852 (95% CI: 0.69–1.00; *p* < 0.001), respectively (Figure 1c). Among these markers, AAR showed moderate performance, whereas AAPRI, API, APRI, FIB-4, GPR, and S-Index demonstrated good performance. For HBeAg-positive patients, FIB-4 0.873 and S-Index 0.828 (95% CI: 0.72–0.93; *p* = 0.076) showed good performance, while GPR 0.779 (95% CI: 0.65–0.90; *p* = 0.183) showed moderate performance. In HBeAg-negative patients, FIB-4 0.862 (95% CI: 0.65–1.00; *p* = 0.001), S-Index 0.856 (95% CI: 0.65–1.00; *p* = 0.001), and GPR 0.830 (95% CI: 0.62–1.00; *p* = 0.002) all showed good performance. The cut-off values calculated for AAPRI, AAR, API, APRI, FIB-4, GPR, and S-Index were 0.005, 0.805, 2.50, 0.835, 2.145, 0.575, and 0.129, respectively. The diagnostic performance of non-invasive fibrosis markers and the cut-off values with sensitivities and specificities for cirrhosis are presented in Table 5.

We found that, except for AAR, all of the other markers could differentiate between significant fibrosis from non-significant fibrosis and cirrhosis from non cirrhosis (*p* > 0.005). Among HBeAg-positive patients, FIB-4 had the highest AUROC for significant fibrosis (0.817) and cirrhosis (0.873), while GPR (0.873) performed the best for advanced fibrosis; in HBeAg-negative patients, FIB-4 had the highest AUROC for advanced fibrosis (0.848) and cirrhosis (0.862), whereas GPR (0.713) demonstrated the strongest accuracy for significant fibrosis.

## 4. Discussion

Despite its continued use, liver biopsy remains an imperfect reference standard. It is invasive, costly, carries a risk of complications, is poorly accepted by patients, requires expert interpretation, and suffers from interobserver and sampling variability. Consequently, current guidelines recommend liver biopsy only when non-invasive markers provide indeterminate results in the management of CHB. On 2 November 2024, the Turkish Social Security Institution published a revised edition of the Health Implementation Communique on Hepatitis B, which recognized the non-invasive markers APRI and FIB-4 as acceptable criteria for initiating antiviral therapy [18]. Therefore, the use of these markers is expected to expand, supporting clinical decision-making and reducing unnecessary liver biopsies [19,20,21].

In this study, we evaluated the diagnostic performance of seven non-invasive fibrosis markers against liver biopsy in treatment-naive patients with CHB. Among them, the GGT-to-platelet ratio (GPR) is a more recently developed non-invasive fibrosis marker than APRI and FIB-4 for patients with CHB. Ekin et al. reported moderate accuracy of GPR for significant (AUROC: 0.721) and advanced (AUROC: 0.796) fibrosis, and high accuracy for cirrhosis (AUROC: 0.851) [22]. Similarly, a meta-analysis by Lian et al. [23] confirmed its moderate accuracy for predicting significant fibrosis (AUROC: 0.733), advanced fibrosis (AUROC: 0.777), and cirrhosis (AUROC: 0.796). Consistent with these data, our findings show that GPR had moderate (AUROC: 0.719) accuracy for significant fibrosis and high accuracy for advanced fibrosis (AUROC: 0.838) and cirrhosis (AUROC: 0.826). Furthermore, in a study by Liu et al. [24], GPR was reported to demonstrate the best performance across all stages of fibrosis in both HBeAg-positive and HBeAg-negative patients, which is consistent with our findings.

Regarding APRI, previous studies showed inconsistent results. Liao et al. [25] reported AUROC values of 0.760, 0.740, and 0.770 for significant fibrosis, advanced fibrosis, and cirrhosis, respectively, suggesting moderate accuracy. Kang et al. [26] reported comparatively lower values of 0.680, 0.757, and 0.678. In our study, APRI demonstrated poor diagnostic accuracy for significant fibrosis (AUROC: 0.664) but moderate diagnostic accuracy for advanced fibrosis (AUROC: 0.797) and good diagnostic accuracy (AUROC: 0.811) for cirrhosis. According to World Health Organization guidelines, an APRI cut-off > 0.5 should be used for the assessment of significant fibrosis, while a cut-off > 1.0 for cirrhosis [27]. In our study, the cut-offs were >0.55 for significant fibrosis (sensitivity: 66.7%; specificity: 61.3%; *p* < 0.001) and >0.83 (sensitivity: 80%; specificity: 74.9%; *p* < 0.001) for cirrhosis. The cut-off value for APRI score was found to be 0.98 in HBeAg-positive patients and 0.57 in HBeAg-negative patients (*p* < 0.05) in our study. Similarly, Doğan et al. [28], reported APRI ≥ 0.358, with sensitivity of 72.2% specificity of 73.7% (*p* > 0.05). In contrast, Kaya et al. [29] found a lower sensitivity 59.64% for APRI at cut-offs of ≥16.24 with specificity of 73.61% (*p* < 0.05) for significant fibrosis. Differences between studies may reflect variations in cut-off thresholds and patient populations, as well as differences in HBeAg status distribution.

FIB-4 was initially validated for determining advanced fibrosis in people co-infected with HIV and HCV [20], and has shown variable diagnostic performance across studies. The reported AUROCs ranged from 0.593/0.674/0.671 in Zhao et al. [30] to 0.703/0.680/0.617 in Tag-Adeen et al. [31] for significant fibrosis, advanced fibrosis, and cirrhosis, respectively. In contrast, our findings (0.717/0.800/0.865) indicate a comparatively stronger performance. Differences in results between studies can be attributed to age distribution, and ALT fluctuations.

According to our findings, AAR showed poor performance in significant (AUC: 0.540) and advanced fibrosis (AUC: 0.540) while showing moderate performance in cirrhosis (AUC: 0.748). These results are consistent with other studies. Wang et al. [32] reported AUROC values of 0.52 for significant fibrosis and advanced fibrosis. In another study, Tag-Adeen et al. [31] reported AUROC values of 0.596, 0.665, and 0.670 for significant fibrosis, advanced fibrosis, and cirrhosis emphasizing the limited value of AAR. Importantly, our analysis revealed that, with the exception of AAR, all other evaluated noninvasive markers may accurately differentiate significant from non-significant fibrosis and cirrhosis from non-cirrhosis (*p* > 0.05). Overall, these findings suggest that AAR may provide some accuracy in cirrhosis, but its role in detecting significant and advanced fibrosis remains limited.

For the first time, Poynard et al. evaluated API as a model for fibrosis in CHC patients [15]. This model has been shown to be a good predictive index for liver fibrosis stage in many studies. Wang et al. [32] reported, in their study, AUROC values of API 0.679 for significant fibrosis and 0.759 for advanced fibrosis. In a study by Korkmaz et al. [33], AUROC values were reported as API 0.629 for significant fibrosis and 0.775 for cirrhosis. In the present study, AUROC for API was 0.661, 0.85, and 0.80 for significant fibrosis, advanced fibrosis, and cirrhosis, respectively. The AUROC value for significant fibrosis is consistent with the literature, but for advanced fibrosis and cirrhosis, our values are higher.

The S-index has a higher diagnostic accuracy for CHB, particularly for the detection of advanced fibrosis and cirrhosis. We found the AUROC values for significant fibrosis, advanced fibrosis, and cirrhosis were 0.712, 0.836, and 0.852, respectively. In a Turkish cohort, Sayar et al. [34] reported a lower performance for significant fibrosis (AUROC: 0.683), but a higher accuracy for cirrhosis. Recently, Guo et al. [35] reported AUROC values for significant fibrosis (0.791), advanced fibrosis (0.867), and cirrhosis (0.961). The S-index might have poor sensitivity in the early stages, but it gains diagnostic strength as fibrosis progresses and the biochemical parameters used in the formula become more significantly altered.

There is limited data about the AUROC values of AAPRI. According to our findings, it has limited diagnostic accuracy for liver fibrosis. In our study, AAPRI showed poor performance in significant (AUC: 0.590) and advanced fibrosis (AUC: 0.723) while showing moderate performance in cirrhosis (AUC: 0.850). In a study by Ekin et al. [22], the authors reported AUROC values of AAPRI 0.566, 0.672, and 0.792 for significant fibrosis, advanced fibrosis, and cirrhosis. In another study, Sha et al. [36] reported AUROC values of 0.531 for significant fibrosis.

Therefore, these differences between studies may be attributed to variability in different standards of ALT levels, patient populations, the distribution of fibrosis stages, the proportion of advanced fibrosis, the reference standards applied, and the cut-off thresholds used, in addition to laboratory and methodological heterogeneity. APRI, GPR, FIB-4, and S-Index were found to perform better than other non-invasive markers for determining advanced liver fibrosis and cirrhosis in our study; their sensitivity and specificity were limited for significant fibrosis. Hu et al. [37] reported that the combination of the GPR and APRI or the GPR andFIB-4 showed higher AUROC values than single indices, especially for detecting cirrhosis. Ayed et al. [38] combined two non-invasive markers (APRI and FIB-4) for predicting liver fibrosis in patients and revealed better predictive performance in comparison with APRI and FIB-4 scores tested alone. Thus, combining these markers (APRI, GPR, FIB-4, and S-Index) may enhance accuracy in detecting liver fibrosis and cirrhosis, which may reduce the need for liver biopsy in CHB patients.

## 5. Limitations

This study has several limitations. First, its retrospective single-center design may have limited the generalizability of the findings. Second, the number of patients with advanced fibrosis was relatively low. This may be explained by national reimbursement policies, under which liver biopsy is not mandatory for patients with compensated cirrhosis to initiate antiviral treatment. Non-invasive methods for fibrosis assessment, such as liver stiffness measurement by elastography and serum-based tests, are generally preferred over liver biopsy for evaluating liver fibrosis and monitoring its progression. However, elastography was not available in our center during the study period. A notable strength of this study is the assessment of the diagnostic performance of non-invasive fibrosis markers according to the HBeAg status of patients with significant fibrosis, advanced fibrosis, and cirrhosis. The exclusive use of non-invasive fibrosis markers is frequently met with caution by clinicians, primarily due to concerns about their diagnostic precision and reliability in comparison with the gold-standard liver biopsy. Therefore, this study contributes to the existing literature by evaluating and comparing the diagnostic performance of seven non-invasive fibrosis markers against liver biopsy findings in patients with CHB.

## 6. Conclusions

In conclusion, FIB-4, GPR, and S-Index were useful for assessing all stages of liver fibrosis, including significant fibrosis, advanced fibrosis, and cirrhosis. The clinical applicability of AAR was limited, since it could not accurately differentiate significant fibrosis from non-significant fibrosis and cirrhosis from non-cirrhosis. These findings highlight the value of non-invasive markers as inexpensive and easily applicable methods for clinicians in assessing the stage of liver fibrosis. These advantages are expected to encourage the growing clinical use of non-invasive markers and overall improve patient outcomes. Since early liver fibrosis may regress or even completely resolve [39], the timely initiation of antiviral therapy is essential. Broader use of these non-invasive markers could therefore facilitate earlier treatment decisions and reduce the need for liver biopsies.

## Figures and Tables

**Table 1:** Demographic and laboratory characteristics of patients according to fibrosis groups.

Variables	F0 (*n* = 14)	F1 (*n* = 67)	F2 (*n* = 364)	F3 (*n* = 64)	F4 (*n* = 13)	F5 (*n* = 3)	F6 (*n* = 11)	*p*-Value
Gender (female/male)	6/8	34/33	138/226	15/49	2/11	1/2	1/10	0.009
HBeAg (−/+)	12/2	52/15	257/96	51/13	7/6	1/2	9/2	0.224
Age (years)	42.2 ± 12.1	44.9 ± 13.8	42.8 ± 12.0	51.7 ± 11.2	54.0 ± 12.7	37.0 ± 12.5	61.0 ± 13.0	<0.001
AST (IU/L)	46.2 ± 23.5	51.5 ± 52.0	42.4 ± 40.5	62 ± 58.1	63.7 ± 42.5	42.0 ± 32.7	81.7 ± 75.4	0.003
ALT (IU/L)	78.7 ± 64.6	72.5 ± 77.9	68.8 ± 86.8	95.8 ± 108	104.6 ± 80.7	63.7 ± 67.6	68.0 ± 46.1	0.320
Albumin (g/L)	43.2 ± 3.7	42.2 ± 4.0	43.4 ± 4.5	42.9 ± 4.6	42.7 ± 4.7	40.5 ± 2.1	32.5 ± 8.2	<0.001
Platelet counts (×10^3^/µL)	224 ± 52.5	243 ± 70.1	224 ± 55.1	198 ± 53.1	180 ± 49.1	244 ± 23.7	123 ± 52.3	<0.001

HBeAg, hepatitis B e antigen; AST, aspartate aminotransferase; ALT, alanine aminotransferase.

**Table 2:** Comparison of the non-invasive markers between fibrosis stages and cirrhosis status.

Non-Invasive Markers	Non-Significant Fibrosis (F0–2) Med (Min–Max)	Significant Fibrosis (F3–6) Med (Min–Max)	*p*-Value	Non-Cirrhotic (F0–4) Med (Min–Max)	Cirrhotic (F5–6) Med (Min–Max)	*p*-Value
AAPRI	0.0033 (0.0009–0.204)	0.0045 (0.0013–0.502)	<0.001	0.0034 (0.0009–0.0204)	0.0065 (0.0025–0.0502)	<0.001
AAR	0.76 (0.29–2.67)	0.78 (0.29–1.79)	0.564	0.75 (0.29–2.67)	1.07 (0.54–1.73)	0.005
API	1 (0–12)	2 (0–13)	<0.001	1 (0–12)	3 (1–13)	<0.001
APRI	0.44 (0.12–5.92)	0.83 (0.16–6.54)	<0.001	0.465 (0.12–6.54)	1.15 (0.17–5.54)	0.004
FIB-4	0.94 (0.27–7.75)	1.62 (0.54–21.32)	<0.001	1 (0.27–8.73)	2.81 (0.54–21.32)	0.001
GPR	0.29 (0.06–3.58)	0.515 (0.08–4.98)	<0.001	0.3 (0.06–3.58)	0.715 (0.11–4.98)	0.001
S-Index	0.053 (0.011–1.906)	0.094 (0.014–3.113)	<0.001	0.057 (0.011–1.906)	0.264 (0.023–3.113)	<0.001

AAR, AST-to-ALT ratio; AAPRI, AAR-to-platelet ratio index; APRI, AST-to-platelet ratio index; API, age-platelet index; FIB-4, fibrosis-4 index; GPR, GGT-to-platelet ratio index; Med, median; min, minimum; max, maximum.

**Table 3:** Diagnostic performance of non-invasive fibrosis markers in treatment-naive patients with significant fibrosis.

		AUC	95% CI		*p*-Value	Cut-Off	Sensitivity	Specificity	Youden Index
AAPRI	Total	0.590	0.48	0.69	0.067	0.004	57.1	59.2	16.3
	HBe Ag +	0.588	0.34	0.82	0.407	0.003	55.6	56.8	12.4
	HBe Ag −	0.582	0.45	0.70	0.145	0.004	56.3	65.7	22
AAR	Total	0.540	0.43	0.64	0.421	0.805	57.1	52.9	10
	HBe Ag +	0.593	0.36	0.82	0.381	0.655	55.6	56.8	12.4
	HBe Ag −	0.513	0.39	0.63	0.814	0.885	50	58.7	8.7
API	Total	0.661	0.56	0.75	0.001	1.5	69	57.6	26.6
	HBe Ag +	0.674	0.50	0.84	0.102	1.5	88.9	45.5	34.4
	HBe Ag −	0.666	0.55	0.77	0.003	1.5	65.6	61.5	27.1
APRI	Total	0.664	0.57	0.75	0.001	0.555	66.7	61.3	28
	HBe Ag +	0.606	0.41	0.79	0.320	0.980	55.6	68.2	23.8
	HBe Ag −	0.676	0.56	0.78	0.002	0.575	62.5	66.4	28.9
FIB-4	Total	0.717	0.63	0.80	<0.001	1.17	69	64.9	33.9
	HBe Ag +	0.817	0.68	0.94	0.003	1.140	77.8	79.5	57.3
	HBe Ag −	0.679	0.57	0.78	0.002	1.205	65.6	62.9	28.5
GPR	Total	0.719	0.63	0.80	<0.001	0.445	69	74.3	43.3
	HBe Ag +	0.799	0.68	0.91	0.005	0.475	77.8	75	52.8
	HBe Ag −	0.713	0.60	0.81	<0.001	0.445	68.8	74.1	42.9
S-Index	Total	0.712	0.62	0.80	<0.001	0.075	66.7	67	33.7
	HBe Ag +	0.803	0.68	0.92	0.004	0.093	77.8	75	52.8
	HBe Ag −	0.702	0.59	0.80	<0.001	0.064	75	61.5	36.5

AUC, area under the curve; AAR, AST-to-ALT ratio; AAPRI, AAR-to-platelet ratio index; APRI, AST-to-platelet ratio index; API, age-platelet index; FIB-4, fibrosis-4 index; GPR, GGT-to-platelet ratio index; ALT, alanine aminotransferase; AST, aspartate aminotransferase; GGT, γ-glutamyl transferase.

**Table 4:** Diagnostic performance of non-invasive fibrosis markers in treatment-naive patients with advanced fibrosis.

		AUC	95% CI		*p*-Value	Cut-Off	Sensitivity	Specificity	Youden Index
AAPRI	Total	0.723	0.54	0.89	0.005	0.004	71.4	70.8	42.2
	HBe Ag +	0.506	0.17	0.83	0.964	0.002	80	29.2	9.2
	HBe Ag −	0.861	0.74	0.98	<0.001	0.005	77.8	75.3	53.1
AAR	Total	0.540	0.43	0.64	0.421	0.805	57.1	52.9	10
	HBe Ag +	0.481	0.21	0.74	0.891	0.625	60	50	10
	HBe Ag −	0.738	0.54	0.93	0.016	1.135	77.8	80	57.9
API	Total	0.805	0.69	0.91	<0.001	2.50	57.1	82.2	39.3
	HBe Ag +	0.750	0.57	0.92	0.068	2.50	60	72.9	32.9
	HBe Ag −	0.809	0.65	0.96	0.002	2.50	55.6	84.3	39.9
APRI	Total	0.797	0.65	0.94	<0.001	0.835	78.6	75.8	54.4
	HBe Ag +	0.654	0.39	0.91	0.260	0.980	80	68.8	48.8
	HBe Ag −	0.822	0.62	1.00	0.001	0.770	88.9	77.1	66
FIB-4	Total	0.800	0.65	0.94	<0.001	1.445	78.6	75.3	53.9
	HBe Ag +	0.754	0.54	0.96	0.063	1.380	60	81.3	41.3
	HBe Ag −	0.848	0.66	1.00	<0.001	1.445	88.9	73.5	62.4
GPR	Total	0.838	0.71	0.96	<0.001	0.525	85.7	78.5	64.2
	HBe Ag +	0.873	0.77	0.97	0.006	0.550	80	81.3	61.3
	HBe Ag −	0.816	0.63	0.99	0.001	0.575	77.8	82.5	60.3
S-Index	Total	0.836	0.71	0.96	<0.001	0.125	78.6	83.1	61.7
	HBe Ag +	0.850	0.73	0.96	0.011	0.125	80	83	63.3
	HBe Ag −	0.826	0.63	1.00	0.001	0.136	77.8	85.5	63.3

AUC, area under the curve; AAR, AST-to-ALT ratio; AAPRI, AAR-to-platelet ratio index; APRI, AST-to-platelet ratio index; API, age-platelet index; FIB-4, fibrosis-4 index; GPR, GGT-to-platelet ratio index; ALT, alanine aminotransferase; AST, aspartate aminotransferase; GGT, γ-glutamyl transferase.

**Table 5:** The diagnostic performance of non-invasive fibrosis markers in treatment-naive patients with cirrhosis.

		AUC	95% CI		*p*-Value	Cut-Off	Sensitivity	Specificity	Youden Index
AAPRI	Total	0.850	0.70	0.99	<0.001	0.005	80	78	58
	HBe Ag +	0.618	0.17	1.00	0.575	0.006	50	92.2	42.2
	HBe Ag −	0.813	0.83	0.99	<0.001	0.005	87.5	75.4	62.9
AAR	Total	0.748	0.58	0.91	0.008	0.805	57.1	52.9	10
	HBe Ag +	0.520	0.29	0.74	0.926	0.760	50.	64.7	14.7
	HBe Ag −	0.820	0.68	0.95	0.002	1.135	87.5	80.2	67.7
API	Total	0.800	0.65	0.94	0.001	2.50	60	81.6	41.6
	HBe Ag +	0.667	0.45	0.87	0.427	2.50	50	70.6	20.6
	HBe Ag −	0.820	0.65	0.98	0.002	4.50	62.5	94.6	57.1
APRI	Total	0.811	0.63	0.98	0.001	0.835	80	74.9	54.9
	HBe Ag +	0.706	0.57	0.83	0.327	1.145	50	74.5	24.5
	HBe Ag −	0.815	0.59	1.00	0.003	0.770	87.5	76.6	64.1
FIB-4	Total	0.865	0.70	1.00	<0.001	2.145	80	90.6	70.6
	HBe Ag +	0.873	0.74	0.99	0.076	2.500	50	94.1	44.1
	HBe Ag −	0.862	0.65	1.00	0.001	2.145	87.5	89.8	77.3
GPR	Total	0.826	0.66	0.99	<0.001	0.575	80	82.1	62.1
	HBe Ag +	0.779	0.65	0.90	0.183	0.645	50	82.4	32.4
	HBe Ag −	0.830	0.62	1.00	0.002	0.575	87.5	82.6	70.1
S-Index	Total	0.852	0.69	1.00	0.000	0.129	80	85.2	65.2
	HBe Ag +	0.828	0.72	0.93	0.118	0.129	50	84.3	34.5
	HBe Ag −	0.856	0.65	1.00	0.001	0.136	87.5	85.6	73.1

AUC, area under the curve; AAR, AST-to-ALT ratio; AAPRI, AAR-to-platelet ratio index; APRI, AST-to-platelet ratio index; API, age-platelet index; FIB-4, fibrosis-4 index; GPR, GGT-to-platelet ratio index; ALT, alanine aminotransferase; AST, aspartate aminotransferase; GGT, γ-glutamyl transferase.

## Data Availability

The datasets generated during and/or analyzed during the current study are available from the corresponding author upon reasonable request.

## Abbreviations

The following abbreviations are used in this manuscript:

AAR	AST-to-ALT Ratio
AAPRI	AAR-to-Platelet Ratio Index
ALT	Alanine Aminotransferase
APRI	AST-to-Platelet Ratio Index
API	Age-Platelet Index
AST	Aspartate Aminotransferase
AUC	Area Under the Curve
AUROC	Area Under the Receiver Operating Characteristic Curve
CHB	Chronic Hepatitis B
CHC	Chronic Hepatitis C
CI	Confidence Interval
FIB-4	Fibrosis-4 Index
GGT	γ-Glutamyl Transpeptidase
GPR	γ-Glutamyl Transpeptidase-to-Platelet Ratio
HAI	Histology Activity Index
HBV	Hepatitis B Virus
HCV	Hepatitis C Virus
HDV	Hepatitis D Virus
HIV	Human Immunodeficiency Virus
IQR	Interquartile Range
PLT	Platelet Count
ROC	Receiver Operating Characteristic
S-Index	Serum Index (1000 × GGT)/(PLT × Albumin^2^)
SD	Standard Deviation
